# Biventricular Heart Failure With Reduced Ejection Fraction in a Young Male: A Case Report

**DOI:** 10.7759/cureus.108285

**Published:** 2026-05-05

**Authors:** Chirag Lodha, Eric J Basile

**Affiliations:** 1 Internal Medicine, University of South Florida Morsani College of Medicine, Tampa, USA; 2 Cardiovascular Disease, University of South Florida Morsani College of Medicine, Tampa, USA

**Keywords:** biventricular dysfunction, biventricular failure, heart failure, heart failure with reduced ejection fraction, icd

## Abstract

A 33-year-old male with a past medical history of nonischemic cardiomyopathy with a reduced ejection fraction (HFrEF) and hypertension presented with worsening dyspnea and chest pain. This had started over the past three days but had not improved, prompting him to come to the emergency department. His vitals were unremarkable on presentation. Initial evaluation due to his history of HFrEF was obtaining an echocardiogram, which revealed an ejection fraction of 15%-20%, similar to the previous echocardiogram. He underwent cardiac MRI later, which showed an ejection fraction of 6% for the left ventricle and 17% for the right ventricle. Multidisciplinary teams optimized guideline-directed medical therapy (GDMT) during this hospitalization, including uptitration of sacubitril-valsartan, metoprolol succinate, spironolactone, and initiation of empagliflozin and digoxin. Right heart catheterization guided management, and a wearable defibrillator bridged to future implantable cardioverter-defibrillator placement. Severe obstructive sleep apnea was diagnosed and treated with continuous positive airway pressure (CPAP). The patient was discharged home in stable condition after one week with an optimized regimen, with advanced therapy evaluation including transplant workup. This case highlights GDMT optimization in young patients with advanced HFrEF, potential for recovery, and bridging strategies, addressing a complex and tenuous disease status in an increasingly more common patient population.

## Introduction

Nonischemic cardiomyopathy (NICMO) in young adults frequently progresses to severe heart failure with reduced ejection fraction (HFrEF), necessitating aggressive guideline-directed medical therapy (GDMT) and early evaluation for advanced therapeutic interventions. Patients under the age of 40 with a nonischemic etiology often demonstrate significantly higher rates of left ventricular functional recovery when combining GDMT with advanced mechanical circulatory support (MCS) therapies [1]. GDMT typically involves a combination of renin-angiotensin system inhibitors, beta-blockers, mineralocorticoid receptor antagonists, and sodium-glucose cotransporter-2 inhibitors (SGLT2is), all of which have been shown to substantially reduce mortality and hospitalization rates in both clinical trials and real-world practice [2,3]. Despite these benefits, the practical implementation of GDMT has significant challenges, including drug-induced hypotension, renal dysfunction, and general intolerance, particularly in patients with advanced heart failure who may already exhibit hemodynamic instability [4]. In these cases, advanced device therapies, such as left ventricular assist devices (LVADs), have shown promise [5,6].

While younger patients often tolerate a more rapid transition to GDMT, allowing them to be supported by all pillars quickly, they often suffer from a higher rate of medication noncompliance, which is also affected by factors such as socioeconomic status and health literacy [7]. In this patient population, one pill combination medication regimens are emerging as a promising new frontier, although studies remain to be done to show whether or not there is a mortality benefit from a simplified medication regimen [7]. Inpatient settings offer a controlled environment where rapid titration of medications can be safely achieved under close hemodynamic monitoring with advanced devices or Swan-Ganz catheters [8]. However, despite all these precautions, having all pillars of GDMT can often have negative psychosocial effects on a patient's perceived quality of life [9]. A multidisciplinary approach, incorporating heart failure specialists, cardiologists, pharmacists, social workers, and nursing teams, has been shown to significantly increase adherence to GDMT protocols and optimize therapeutic efficacy, especially when utilizing advanced practice practitioners [10]. This, in conjunction with HFrEF education programs, has shown increasing promise in encouraging adherence to GDMT [11]. Bridging strategies, such as implantable cardioverter-defibrillators (ICDs) or LVADs, play a critical role in preventing sudden cardiac death and providing MCS while awaiting potential myocardial recovery or transplantation [12-14]. In some cases of MCS, there is a degree of myocardial recovery, mediated by offloading strain from the left ventricle, allowing the myocardium to slowly recover function [14]. Wearable ICDs serve as an effective temporary solution, offering protection against life-threatening arrhythmias during the period of GDMT optimization and clinical stabilization [13].

Comorbid conditions, including obstructive sleep apnea, uncontrolled hypertension, and diabetes mellitus, can significantly exacerbate heart failure progression and must be aggressively managed alongside primary therapy to achieve optimal outcomes [1]. Younger patients in particular benefit from a comprehensive, individualized care plan that prioritizes maximal medical and device-based therapy to harness their inherent potential for myocardial recovery before considering irreversible interventions such as heart transplantation [3]. This case underscores the importance of meticulous GDMT optimization, proactive comorbidity management, and strategic bridging therapies in a young male patient presenting with end-stage cardiomyopathy, with existing literature strongly supporting these approaches for improving both survival and quality of life in this vulnerable population [9].

## Case presentation

**Figure 1:**
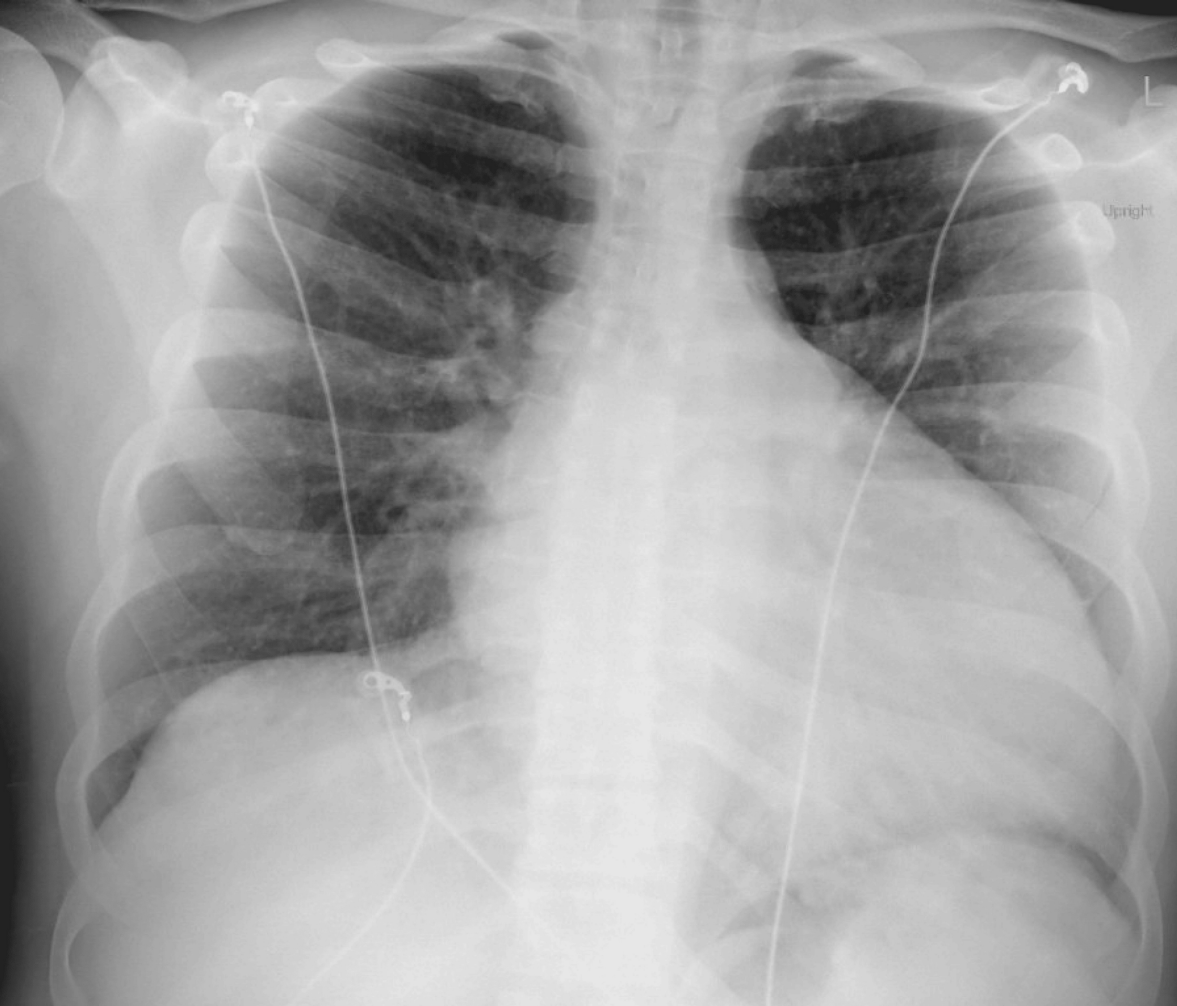
Chest X-ray showing cardiomegaly without effusion.

A 33-year-old male with a past medical history of NICMO, HFrEF, and obstructive sleep apnea, noncompliant on continuous positive airway pressure (CPAP), presented for worsening dyspnea and chest pain for the past three days. He said that the chest pain had no specific triggers, such as exertion, and endorsed exertional dyspnea. His outpatient cardiologist asked for direct admission due to these persistent symptoms, given his history of NICMO. A recent echocardiogram at an outside facility showed an ejection fraction of 15%-20% one month prior. Vital signs were notable for a blood pressure of 105/82 mmHg, a heart rate of 75 beats per minute (BPM), and an oxygen saturation of 98% on room air. Physical examination revealed 2+ pitting edema to the mid-ankles bilaterally, as well as crackles on lung auscultation. Cardiac examination demonstrated a regular rate and rhythm with an audible S3. Labs showed a B natriuretic peptide (BNP) of 200 ng/L, increased from his previous hospitalization over one month ago for similar symptoms. His electrolytes were within normal limits, and his creatinine was at his baseline (1.0 mg/dL). His chest radiograph showed no acute cardiopulmonary process and redemonstrated previously known cardiomegaly, without evidence of effusion or pulmonary edema (Figure 1).

**Figure 2:**
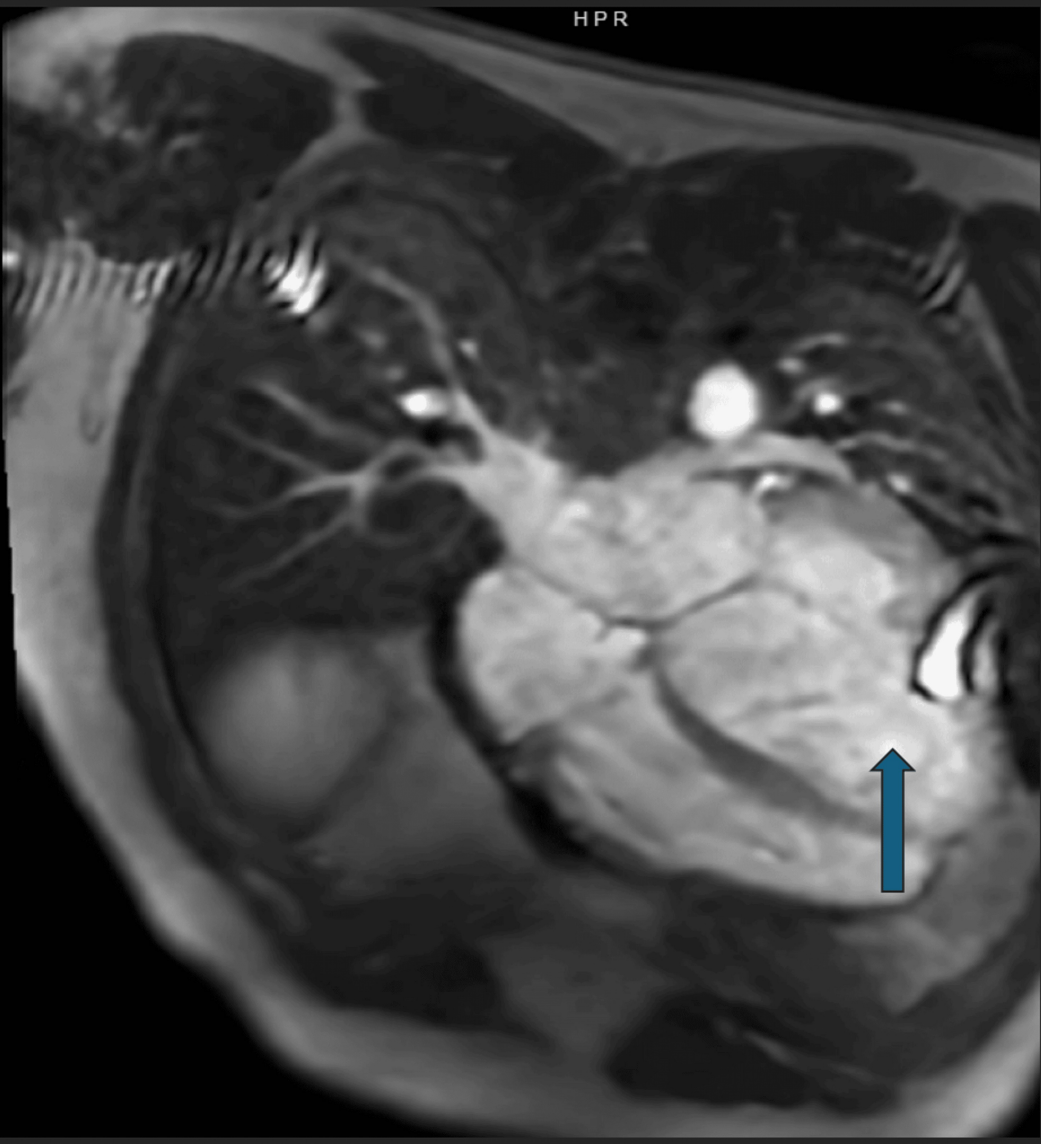
Cardiac MRI demonstrating biventricular failure, with an LVEF of 6% and an RVEF of 17%. The blue arrow denotes a severely dilated left ventricle without regional wall motion abnormalities. LVEF, left ventricular ejection fraction; RVEF, right ventricular ejection fraction

Given his recent exacerbation and need for advanced heart failure management, cardiology, heart failure/transplant, and electrophysiology services were consulted for transplant evaluation. As part of the workup, cardiac MRI demonstrated a severely dilated left ventricle, with a left ventricular ejection fraction (LVEF) of 6% and a right ventricular ejection fraction (RVEF) of 17%, as well as moderate mitral regurgitation and a small pericardial effusion, without evidence of infiltrative disease (Figure 2).

A right heart catheterization performed the same day demonstrated elevated left-sided filling pressures, low cardiac output/index, and increased afterload (Table 1).

**Table 1:** Right heart catheterization values. RA, right atrial

Lab values	Patient value	Normal value
RA pressure (mmHg)	20	8-14
Cardiac Index	2.9	3-5
Afterload (Woods Units)	63.6	<3

A Swan-Ganz catheter was placed for hemodynamic monitoring but was removed the following day after improvement in cardiac output with diuresis. However, given his tenuous hemodynamic status and biventricular failure, he was subsequently transferred to the ICU for close monitoring and GDMT titration. Regimen adjustments increased sacubitril-valsartan, metoprolol succinate, and spironolactone; he was started on empagliflozin and digoxin due to the ionotropic benefit of digoxin therapy. Electrophysiology recommended a wearable cardioverter-defibrillator (LifeVes, ZOLL Medical, Chelmsford, MA) as a bridge to ICD placement, while the heart transplant team deemed him a candidate for heart transplantation, pending further social work assessment and evaluation. During this time, he also underwent a sleep study, which demonstrated severe obstructive sleep apnea, and he was initiated on CPAP therapy. 

He underwent patient education covering heart failure self-management, medication adherence, and daily weight monitoring using a provided scale. One week later, he was transitioned to all four pillars of GDMT and remained hemodynamically stable throughout this process. His discharge medication regimen ultimately included digoxin 125 mcg daily, empagliflozin 10 mg daily, furosemide 20 mg daily, metoprolol succinate 25 mg daily, sacubitril/valsartan 49/51 mg twice daily, and spironolactone 25 mg daily. Follow-up was scheduled with cardiology and electrophysiology for transplant evaluation and ICD placement, which had not yet been completed at the time of writing this case report.

## Discussion

This case demonstrates the successful optimization of GDMT during an inpatient hospitalization for advanced nonischemic HFrEF, which aligns with recent registry data showing the feasibility of achieving comprehensive treatment targets even in patients with severe disease presentations, as highlighted in the literature [2]. Younger age appears to be a favorable factor for myocardial recovery, as multiple studies have reported significantly higher rates of functional improvement in patients with nonischemic HFrEF under the age of 40 compared with older cohorts, suggesting a unique opportunity for intervention in this demographic [1,13]. The uptitration to quadruple therapy, including an angiotensin receptor-neprilysin inhibitor (ARNI), beta-blocker, MRA, and SGLT2i, closely aligns with evidence-based combinations shown to improve survival [3].

The incorporation of multidisciplinary input, including cardiology, pharmacy, and nursing teams, mirrors institutional strategies that have been proven to boost prescription rates and adherence to GDMT by addressing barriers such as medication access, side effect management, and patient education [7,11]. The use of a LifeVest wearable cardioverter-defibrillator as a bridging strategy successfully mitigated arrhythmic risk during the critical period of GDMT optimization, providing a safety net while awaiting further assessment for permanent ICD placement [14]. Plans for advanced therapies, including LVAD or transplant evaluation, were proactively discussed to address potential disease progression, reflecting current clinical trial data comparing the role of LVADs to maximal GDMT in patients who may not initially qualify for definitive interventions [12]. The identification and treatment of sleep apnea as a key precipitant of decompensation further reduced the risk of future hospitalizations, underscoring the importance of addressing modifiable contributors to heart failure exacerbations [15]. Addressing this comorbidity is particularly important, as untreated sleep apnea has been linked to a higher rate of graft failure after cardiac transplant [15].

While managing HFrEF can be multifactorial and complex, additional therapies should also be considered in these patients, who may be tolerating maximum doses of GDMT without much of a clinical response [2]. The definitive treatment for patients with a non-ischemic cardiomyopathy who are not responding to GDMT is a heart transplant. These transplants often last for 12 years on average, which, depending on a patient's age at the time of transplant, can give them close to a normal lifespan [13]. However, in an effort to prolong these patients’ lives, MCS should be considered. This often acts as a bridge to transplant; however, some studies have shown similar efficacy in terms of morbidity and mortality between LVAD and transplant patients [16]. Therefore, in patients with NICMO, LVADs and mechanical support should be considered not only as a bridge to transplant, but also as a potential emerging destination therapy, allowing patients to lead an improved quality of life while awaiting transplant [16].

## Conclusions

In young patients with NICMO, it is important to initiate GDMT promptly, as these patients often demonstrate some degree of improvement in ejection fraction. This, in tandem with MCS devices, has also shown promise in helping with myocardial recovery. The ultimate goal for these patients is a curative one with a heart transplant; however, these can be extremely difficult to obtain, as there is a lack of donor hearts, and this can often take several years, which some patients may not have. Therefore, MCS with LVADs, as well as other devices, should be considered as a bridging therapy for patients with NICMO in addition to GDMT and have shown similar outcomes to heart transplant therapy in some studies. This case illustrates a complex diagnosis in a young patient without cardiovascular risk factors, the addition of GDMT to help prolong this patient's life and discusses additional therapies to help improve morbidity and mortality.
